# Mechanisms of Antibiotic Resistance in Important Gram-Positive and Gram-Negative Pathogens and Novel Antibiotic Solutions

**DOI:** 10.3390/antibiotics10040415

**Published:** 2021-04-10

**Authors:** Loukas Kakoullis, Eleni Papachristodoulou, Paraskevi Chra, George Panos

**Affiliations:** 1 Department of Respiratory Medicine, University General Hospital of Patras, 26504 Patras, Greece; med5796@upnet.gr; 2 Department of Medicine, School of Health Sciences, University of Patras, 26504 Patras, Greece; med6255@upnet.gr; 3 Department of Microbiology, Evangelismos Hospital, 10676 Athens, Greece; pchra@med.uoa.gr; 4 Department of Internal Medicine, Division of Infectious Diseases, University General Hospital of Patras, 26504 Patras, Greece

**Keywords:** *Escherichia coli*, *Staphylococcus aureus*, *Pseudomonas aeruginosa*, *Enterococcus faecalis*, *Enterococcus faecium*, *Acinetobacter baumannii*, *Klebsiella pneumoniae*, MRSA, VRE, multi-drug resistant infections

## Abstract

Multidrug-resistant bacteria have on overwhelming impact on human health, as they cause over 670,000 infections and 33,000 deaths annually in the European Union alone. Of these, the vast majority of infections and deaths are caused by only a handful of species—multi-drug resistant *Escherichia coli*, *Staphylococcus aureus*, *Pseudomonas aeruginosa*, *Enterococcus* spp., *Acinetobacter* spp. and *Klebsiella pneumoniae*. These pathogens employ a multitude of antibiotic resistance mechanisms, such as the production of antibiotic deactivating enzymes, changes in antibiotic targets, or a reduction of intracellular antibiotic concentration, which render them insusceptible to multiple antibiotics. The purpose of this review is to summarize in a clinical manner the resistance mechanisms of each of these 6 pathogens, as well as the mechanisms of recently developed antibiotics designed to overcome them. Through a basic understanding of the mechanisms of antibiotic resistance, the clinician can better comprehend and predict resistance patterns even to antibiotics not reported on the antibiogram and can subsequently select the most appropriate antibiotic for the pathogen in question.

## 1. Introduction

The burden of antimicrobial resistance (AMR) is overwhelming, as it is estimated that over 670,000 infections by AMR pathogens occur annually in the European Union (EU) alone, claiming more than 33,000 lives. The economic impact of these infections is also significant, as the cost to the EU healthcare systems is estimated to exceed 1 billion euros [1]. The vast majority of deaths are caused by only a handful of species: multi-drug resistant (MDR) *Escherichia coli*, *Staphylococcus aureus*, *Pseudomonas aeruginosa*, *Klebsiella pneumoniae*, *Acinetobacter* spp. and *Enterococcus* spp. [2]. The impact of each individual bacterium is summarized in Table 1.

**Figure 1:**
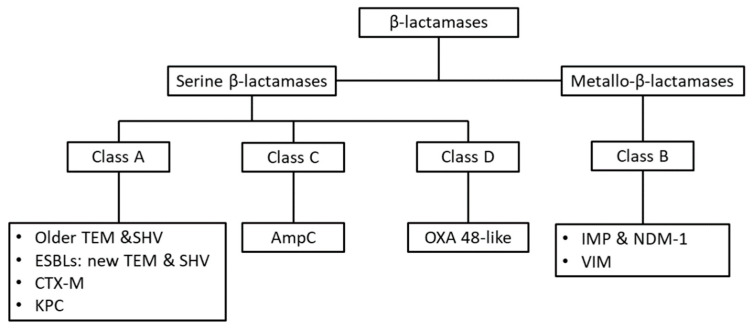
The Ambler classification of β-lactamases, which is based on each enzyme’s primary protein structure. The active site of enzymes of Classes A, C, D contains a serine residue, which is necessary for the hydrolysis of the beta-lactam ring, while enzymes of Class B require zinc ion cofactor in order to function (thus termed metallo-β-lactamases) [3,4]. Abbreviations: ESBL: extended spectrum β-lactamase; TEM, Temoniera; SHV, sulfhydryl variable; CTX-M, Cefotaxime β-lactamase; KPC, *Klebsiella pneumoniae* Carbapenemase; OXA, oxacillinase; IMP, Imipenemase type carbapenemase; NDM-1, New Delhi metallo-β-lactamase; VIM, Verona integron-encoded metallo-β-lactamase.

Resistance mechanisms in these bacteria have evolved rapidly, owing to the presence of selective pressures. Their defense mechanisms against antibiotics involve the production of antibiotic deactivating enzymes, such as the several classes of β-lactamases (Figure 1) or aminoglycoside modifying enzymes, changes in antibiotic targets, and reduction of intracellular antibiotic concentration, either by limiting the entrance of the antibiotic or facilitating its expulsion.

Due to the devastating results of infections caused by these pathogens, appropriate management of such cases is essential. For the clinician, it is important to know and understand the mechanisms of resistance employed by these pathogens, in order to select appropriate antibiotic treatment, especially in cases were the pathogen is known but the antibiogram is still pending. The purpose of this review is to provide an overview of the major mechanisms of resistance utilized by AMR pathogens of great clinical importance, that is *S. aureus*, *Enterococcus* spp., *E. coli*, *P. aeruginosa*, *Acinetobacter* spp. and *K. pneumoniae*.

## 2. Mechanisms of Antimicrobial Resistance in *S. aureus*

*S. aureus* is one of the most significant pathogens in terms of antibiotic resistance, as it has been able to develop resistance mechanisms to nearly all antibiotics used against it. Indeed, from the early 1940′s, when penicillin resistance in *S. aureus* was initially described, *S. aureus* has steadily acquired new mechanisms of resistance, allowing it to become resistant to all β-lactams, tetracyclines, aminoglycosides, fluoroquinolones, clindamycin, trimethoprim-sulfamethoxazole, vancomycin, daptomycin and linezolid, summarized in Table 2 [5,6,7]. Methicillin resistant *S. aureus* (MRSA), the predominant antibiotic resistant strain of *S. aureus*, was estimated to be responsible for over 148,000 infections and 7000 deaths in the EU in 2015 [2].

The initial resistance of *S. aureus* to β-lactams occurred through the development of β-lactamases. The target of β-lactam antibiotics is the transpeptidase moiety in penicillin binding protein (PBP)-2. Β-lactams act as structural analogues of D-Ala4-D-Ala5 and bind to a serine residue in the active site of transpeptidase. They form a stable intermediate (penicilloyl-O-serine) at the active site of the enzyme, which takes up to 4 h to be hydrolyzed, during which time transpeptidase cannot proceed with peptidoglycan synthesis. Serine β-lactamases of *S. aureus* bind to β-lactams and form the same intermediate as formed between β-lactams and transpeptidase. However, the intermediate is quickly hydrolyzed, regenerating the serine of the β-lactamase and releasing penicilloic acid, an inactive degradation product with a broken β-lactam ring. The gene encoding the β-lactamase is located on a trasposome, which can be found either in a plasmid or incorporated into the genome of *S. aureus* [5,8].

Development of resistance to methicillin, and subsequently to all β-lactams, in *S. aureus* occurred through the production of PBP2a, a homologue protein of PBP2. PBP2a is not susceptible to β-lactams, because the targeted serine of the active site of PBP2a is located in a deep pocket, which cannot be accessed by the antibiotics. This structural change is so significant because it makes the active site serine inaccessible to all β-lactams, therefore making MRSA resistant to this entire class of antibiotics. The one exception to this rule is the novel 5th generation cephalosporin, ceftaroline fosamile, which was developed specifically to overcome this mechanism. Ceftaroline fosamile acts by binding to an allosteric site on PBP2a, inducing a conformational change that opens up the deep pocket, which allows the antibiotic to access to the active site serine, inhibiting PBP2a [5,9].

Vancomycin resistance in *S. aureus* comes in two forms: vancomycin resistant *S. aureus* (VRSA) and vancomycin intermediate *S. aureus* (VISA). Apart from a few exceptions, vancomycin resistance has arisen in strains of MRSA. It should be noted that the mechanisms of VISA and VRSA are entirely different, and therefore VISA strains cannot gradually progress to become VRSA [10]. However, in the extremely rare case that VISA acquire additional mechanisms of resistance, such as the vanA operon, they may become VRSA, but through a completely different mechanism [11].

VRSA has acquired vanA operon from vancomycin resistant enterococci (VRE), and was first identified in diabetic patients suffering from concomitant infection by the 2 pathogens [12,13]. The vanA operon allows the bacterium to alter the structure of peptidoglycan precursors from D-Ala-D-Ala to D-Ala-D-Lac, which has a significantly reduced affinity to vancomycin. This confers high-level resistance to vancomycin (minimum inhibitory concentration, MIC ≥ 32 μg-mL) [10].

In contrast, VISA strains continue to produce the D-Ala-D-Ala dipeptide but in a different fashion. Intermediate vancomycin resistance in *S. aureus* appears to arise through mutations in regulatory genes, such as *vraSR*, which control the production of key enzymes in the biosynthesis of the cell wall, leading to upregulation of these enzymes. VISA strains have increased the production of peptidoglycan, leading to thicker cell-walls. However, peptidoglycan in these strains is poorly cross-linked, resulting in D-Ala-D-Ala dipeptides that protrude outwards on the surface of the cell wall and act as decoy targets to vancomycin. As a result, vancomycin binds to these moieties instead of the D-Ala-D-Ala dipeptides at the cell membrane level, where it can exert its action. Therefore, VISA strains block the entry of vancomycin through three interconnected mechanisms: the thicker cell wall hinders the entry of the antibiotic, while the decoy D-Ala-D-Ala dipeptides on the surface of the cell wall not only bind vancomycin, but also lead the cell wall becoming clogged with vancomycin, which further inhibits the entry of the antibiotic [10,14]. Indeed, intact vancomycin molecules have been recovered from the cell walls of VISA strains, demonstrating that the antibiotic is sequestered on their surface. VISA strains have an increased MIC for vancomycin, which lies between 8 and 16 μg-mL [15].

Daptomycin is an antibiotic with a unique mechanism of action. It is an anionic molecule, which binds to calcium, forming cationic complexes which oligomerize to form micelles. These daptomycin-calcium complexes in turn bind to the negatively charged phosphatidylglycerol (PG) on the cell membrane, and lead to the formation of transmembrane cation channels. The resulting outpouring of potassium leads to depolarization and cell death [16]. The high affinity of daptomycin to PG is critical to its mechanism of action and selectivity for Gram-positive bacteria, as PG is major component of the Gram-positive plasma membrane. Furthermore, it is also the reason why daptomycin is not effective in pulmonary infections, as PG makes up approximately 10% of lung surfactant, leading to daptomycin inserting directly into the surfactant layer, as it is unable to distinguish between the PG found in the comparatively small surface area of bacteria and that found in the vast layer of surfactant [17].

Resistance to daptomycin is mediated by mechanisms that alter its interaction with PG. Specifically, a gain of function mutation in the multiple peptide resistance factor (mrpF), a protein that adds a positively charged lysine residue to PG. This leads to an increase in lysinylation of PG and a subsequent increase of the cell-surface charge, which repels the positively charged daptomycin-calcium complex and prevents its attachment to the cell-membrane [16]. Reduction in the expression of negatively charged membrane phospholipids, such as cardiolipin, also changes the membrane charge and leads to daptomycin resistance in *S. aureus* [18].

It should be noted that VISA strains also display a degree of resistance to daptomycin as well, as their cell wall has not only increased thickness, but also an increased positive charge. In contrast, daptomycin resistance through *mrpF* mutations causes MRSA strains to become sensitive to β-lactams. That is because the increase lysinylation of PG is associated with a decrease of PrsA on the cell membrane, a lipoprotein chaperone which is essential for the stability of PBP2a. This is the molecular basis behind the synergistic action of daptomycin and β-lactams against MRSA [5].

Macrolides and clindamycin inhibit protein synthesis by binding to the 23S rRNA in the 50S ribosomal subunit. Resistance to macrolides occurs either by ribosomal methylation at the binding site of the antibiotics, or through efflux pumps, which consume ATP in order to expel the antibiotic from inside the cell. Erythromycin ribosomal methylase (*erm*) genes, which also confer resistance to clindamycin, are prevalent among *S. aureus* strains and can be expressed either constitutively or by induction [19]. Considering that clindamycin is one of the antibiotics of choice for patients with MRSA infections treated in community settings, the phenotype of macrolide, lincosamide and streptogramin type B (MLS_B_) resistance is critical to determine whether prescription of clindamycin will lead to therapeutic failure. While constitutively resistant MLS_B_ strains (cMLS_B_) will appear resistant to both erythromycin and clindamycin on an antibiogram, strains with inducible MLS_B_ (iMLS_B_) resistance will appear as resistant to erythromycin but sensitive to clindamycin; however, these strains can develop resistance to clindamycin during treatment. The standard test used to detect iMLS_B_ resistance is the D-test, in which the *S. aureus* isolate is grown in an agar plate containing an erythromycin and a clindamycin diffusion disk. If no growth is observed then the strain is sensitive to both antibiotics, whereas if growth is observed around both disks then the stain is cMLS_B_ positive, which would have also appeared on the antibiogram; if there is growth around the erythromycin disk which extends to the clindamycin zone of inhibition, then the isolate is iMLS_B_ positive and clindamycin should not be used [4,20].

Linezolid is an antibiotic of the oxazolidinone class, which inhibits protein synthesis by binding to the 23S rRNA segment of the 50S ribosomal subunit and inhibits the ribosomal peptidyl transferase center. It is widely used against resistant Gram-positive bacteria, such as MRSA and VRE. *S. aureus* has been found to develop resistance to linezolid by multiple mechanisms: through mutations to the 23S rRNA, which confers significant resistance; by altering modifications to the 23S rRNA which are required for susceptibility to linezolid, such as the inactivation of a methyltransferase which methylates 23S rRNA; and through mutations to the 50S ribosomal L3 protein, which interacts with the ribosomal peptidyl transferase center [21,22].

Tetracyclines are another antibiotic class that target the ribosome, albeit their target is the 30S ribosomal subunit. Resistance to tetracyclines has limited the utility of a significant class of antibiotics; minocycline for instance is an antibiotic with a long half-life, exceptional bioavailability and tissue penetration used for lung and, skin and soft tissue infections. Furthermore, as a highly lipophilic molecule, minocycline can readily cross the blood–brain barrier and accumulate in the cerebrospinal fluid, making it an ideal antibiotic for central nervous system infections. Resistance to tetracyclines in *S. aureus* is mediated either through ribosomal protection proteins, which act to dislodge the tetracycline from its ribosomal binding site, or through the presence of efflux pumps. Tigecycline, a modified version of minocycline, is built in such a manner to overcome these 2 mechanisms. Tigecycline has a 10- to a 100-fold higher affinity for the ribosomal binding site, while its molecule contains bulky substitutions, which prevent efflux pumps from binding the antibiotic. As a result, tigecycline is a potent antibiotic which can be used against MRSA strains resistant to tetracyclines. However, its pharmacokinetic profile differs from that of its parent molecule as, unlike minocycline, the cerebrospinal fluid penetration of tigecycline is limited. It should be noted that for staphylococci, while tigecycline is more active against the methicillin-resistant strains, minocycline remains more active than tigecycline against methicillin-susceptible strains. Furthermore, tigecycline resistance can also develop, through the production of efflux pumps [5,23,24,25,26,27].

Aminoglycosides are another class of antibiotics that bind to the 30S ribosomal subunit and are rapidly bactericidal, due to their ability to induce errors in mRNA translation. While normally the bacterial ribosome may have an error rate of 1–1000 to 1–10,000 amino acids, aminoglycosides increase the error rate to 1–100 amino acids, which translates to the average protein containing approximately 3 mistakes. This has lethal consequences for the bacterium, especially in the case that the disrupted proteins constitute membrane proteins [28]. In *S. aureus*, aminoglycoside resistance is mediated through enzymatic inactivation, specifically through enzymes that acetylate and phosphorylate aminoglycosides [29].

Rifampicin is an antibiotic also used in *S. aureus* infections, especially because of its ability to penetrate into tissues, biofilms and abscesses. It acts by inhibiting the β subunit of the bacterial RNA polymerase. Resistance to rifampicin occurs through mutations in the gene of RNA polymerase, *rpoB*, which lead to amino acid substitutions at the site of rifampicin binding on the RNA polymerase [30].

Finally, *S. aureus* is also resistant to trimethoprim-sulfamethoxazole (TMP-SMX) and fluoroquinolones, two other classes of antibiotics which inhibit DNA synthesis with different mechanisms. The combination of TMP-SMX inhibits dihydropteroate synthase (DHPS) and dihydrofolate reductase (DHFR), sequential enzymes necessary for the synthesis of folate, required in the synthesis of DNA [31]. *S. aureus* resistance to TMP-SMX results from the production of DHPS and DHFR enzymes that contain amino acid substitutions that make them resistant to the antibiotic combination [5]. On the other hand, fluoroquinolones work by inhibiting DNA gyrase and topoisomerase IV, enzymes responsible for regulating the supercoiling of DNA during replication. Inhibition of these enzymes leads to breaks in DNA, due to the buildup of mechanical stress from supercoiling, leading to cell death [31]. In *S. aureus*, resistance to fluoroquinolones is either mediated through efflux pumps or through mutational amino acid substitutions in the fluoroquinolone binding site of topoisomerase IV and DNA gyrase. As staphylococci are very sensitive to fluoroquinolones, mutations in both enzymes are required for resistance to develop [5].

## 3. Mechanisms of Antimicrobial Resistance in *Enterococcus* spp.

*Enterococcus faecium* and *Enterococcus faecalis* are not only pathogens of great clinical importance in regards to antimicrobial resistance, but also lend their abilities to other bacterial species, such as vancomycin resistance to VRSA [12]. Furthermore, in addition to multiple acquired mechanisms of antibiotic resistance, enterococci are also intrinsically resistant to many antibiotics [32]. VRE, the most clinically significant isolates of *E. faecium* and *E. faecalis*, were responsible for over 16,000 infections and 1000 deaths in the EU in 2015 [2]. Mechanisms of resistance in enterococci are summarized in Table 2.

Enterococci are intrinsically resistant to β-lactams, as their PBPs have a low affinity for β-lactam antibiotics. They exhibit different degrees of susceptibility between the different classes of β-lactams: enterococci are most sensitive to penicillin and ampicillin (*E. faecalis* more so than *E. faecium*), less sensitive to carbapenems and completely resistant to cephalosporins. The MICs for penicillin and ampicillin for *E. faecalis* and *E. faecium* are much higher than those of other Gram-positive organisms that do not express low affinity PBPs [32,33,34]. Intrinsic cephalosporin resistance is also likely due to the low affinity of enterococcal PBPs to antibiotics, especially PBP5, as well as other mechanisms such as two-component signal transduction systems [34]. However, paradoxically, the redundant combination of ampicillin and ceftriaxone has been found to be clinically effective against *E. faecalis*, with the combination considered as effective as ampicillin and gentamycin, which is the standard of care [33]. Enterococci can also acquire mutations to their PBPs, which confer high level resistance to penicillins, as is the case with PBP5 of *E. faecium*. In addition, enterococci can acquire plasmid-mediated *bla* genes for β-lactamase production, identical to those described in *S. aureus* [32].

Despite the well-known synergism of aminoglycosides and β-lactams, both *E. faecalis* and *E. faecium* are intrinsically resistant to aminoglycosides, as their cell wall is impenetrable to aminoglycosides. However, in combination with a cell wall synthesis inhibitor such as ampicillin, aminoglycosides can enter the enterococcal cell and exert their bactericidal action [32]. In addition, *E. faecium* is also able to produce acetyltransferases and phosphotransferases which enzymatically inhibit multiple aminoglycosides, such as tobramycin, kanamycin and amikacin. As a result, the only reliable choices against enterococci are gentamycin and streptomycin [33]. However, enterococci can acquire mobile genetic elements that confer high level resistance against these aminoglycosides as well. Acquired resistance against gentamycin is mediated through enzymes that phosphorylate and acetylate the antibiotic, making it unable to bind to the 30S ribosomal subunit. Streptomycin resistance is mediated by aminoglycoside-modifying enzymes, and can also occur through ribosomal mutations, the presence of which increases the MIC for streptomycin 8 times more than that achieved by the production of aminoglycoside-modifying enzymes [32].

Enterococci are also intrinsically resistant to TMP-SMX, due to their unusual ability to absorb folate from their environment. This can lead to enterococci paradoxically appearing susceptible to TMP-SMX in vitro, which however is not translated to clinical effectiveness against enterococci in vivo [32].

Fluroquinolone resistance in enterococci appears to be mostly acquired. Mechanisms include the presence of mutations at the “quinolone resistance-determining regions”, which reduce the affinity of the target enzymes for the antibiotic, the production of efflux pumps, as well as production of a protection protein, encoded by the *qnr* gene, which is thought to protect DNA gyrase by reducing the binding of fluoroquinolones to the enzyme [33].

Enterococci are also intrinsically resistant to macrolides and clindamycin. Macrolide resistance appears to be mediated through efflux pumps and deactivating enzymes, such as an erythromycin methylase encoded by the *erm* gene [35]. The mechanism of clindamycin resistance appears to differ among species. In *E. faecalis*, resistance is based on the presence of the *lsa* gene (named for lincosamide and streptogramin A resistance), which encodes for an ATP-energized efflux pump [36]. In *E. faecium,* resistance appears to be mediated through the *linB* gene, which encodes for a nucleotidyltransferase, an enzyme that deactivates clindamycin through adenylation [37].

Tetracycline resistance has also been noted in enterococci. Mechanisms include the production of efflux pumps and ribosomal protection proteins [38]. Upregulation of genes associated with these mechanisms, such as *tetL* and *tetM*, has been found to lead to development of resistance to tigecycline as well [39].

The mechanism of daptomycin resistance appears to differ among the two species of *Enterococcus*. *E. faecium* appears to use a similar mechanism to that of *S. aureus*, which is based on electrostatic repulsion of the daptomycin-calcium complex from the cell membrane, by increasing the positive charge of the membrane [16]. *E. faecalis* utilizes a different and more complex mechanism than *E. faecium*, which is based on 2 principles: one, that daptomycin-calcium complex preferentially binds to the cell membrane at the division septum plane; and two, that cardiolipin, a cell membrane phospholipid, enables daptomycin to reach the inner layer of the cell membrane, a process that is required for pore formation. Daptomycin resistance occurs when mutations in the LiaFSR signaling system, which controls the homeostasis of the cell membrane and cell wall, leading to the redistribution of cardiolipin from the septum to non-septal locations on the cell membrane. As a result, daptomycin cannot oligomerize in the septal cell membrane but instead is diverted away from the septum [16,33].

The mechanism of vancomycin resistance in enterococci is identical to the one described for VRSA, as it was passed to *S. aureus* by VRE [10]. However, there are different phenotypes of vancomycin resistance among enterococci. Genes encoding for vancomycin resistance are located on operons, of which most common are vanA (the operon also found in VRSA) and vanB. Strains that have the vanA operon are resistant to both vancomycin and teicoplanin, whereas strains with the vanB operon are only resistant to vancomycin, while they retain sensitivity to teicoplanin [40].

Linezolid is one of the cornerstones of treatment for VRE. Inevitably, linezolid resistance has also been noted in enterococci, and is associated with mutations to the 23S rRNA, similar to those described in *S. aureus* [22].

Although rifampicin is not commonly used in the treatment of enterococci, resistance in rifampicin has been noted in both *E. faecium* and *E. faecalis*. Similar to *S. aureus*, resistance to rifampicin occurs through mutations in the RNA polymerase gene, *rpoB* [41].

## 4. Mechanisms of Antimicrobial Resistance in *P. aeruginosa*

*P. aeruginosa* is one of the most significant MDR Gram-negative pathogens, second only to *E. coli* in terms of numbers of infections and deaths. It is estimated that MDR *P. aeruginosa* is responsible for over 72,000 infections and over 4800 deaths in the EU annually. Of these, an estimated 61,892 cases and 4155 deaths are attributed to carbapenem resistant strains, whereas 1262 cases and 84 deaths are attributed to colistin resistant strains [2].

While other bacteria have different intrinsic mechanisms of resistance for several antibiotics, what sets *P. aeruginosa* apart is that each of its intrinsic resistance mechanisms confers resistance to multiple antibiotics at once. The restricted membrane permeability renders *P. aeruginosa* impervious to many antibiotics and its membrane is estimated to be between 12 and 100 times less permeable than that of *E. coli*. The antibiotics that do make it into the *P. aeruginosa* cell, are subject to efflux pumps and antibiotic-inactivating enzymes. In addition, through the acquisition of mutations, *P. aeruginosa* can develop deficiency in porins, which reduce its permeability even further, can make modifications to antibiotic targets or can overexpress efflux pumps and antibiotic-inactivating enzymes. Finally, *P. aeruginosa* has also adaptive mechanisms of antibiotic resistance, such as the generation of biofilm and persister cells. Biofilms consist of bacterial communities that exist within a matrix made up of polysaccharides, extracellular DNA, carbohydrates, proteins and other bacterial components. Bacteria within a biofilm environment are protected from the host immune system as well as from environmental stressors. Persister cells are dormant cells that stop the synthesis of antibiotic target proteins and as a result can withstand high concentrations of antibiotics, despite not being genetically resistant to them [42,43]. Mechanisms of antibiotic resistance in *P. aeruginosa* and other important Gram-negative bacteria are summarized in Table 3.

*P. aeruginosa* has both intrinsic as well as acquired mechanisms of resistance against β-lactams. Intrinsic include influx pumps, as well as several β-lactamases. Specifically, *P. aeruginosa* has chromosomally encoded AmpC β-lactamases and extended-spectrum-β-lactamases (ESBLs). Acquired β-lactam resistance mechanisms in *P. aeruginosa* include mutations in porins, such as deficiency of the OprD porin which leads to high-level resistance to imipenem and other carbapenems; overexpression of hydrolyzing enzymes such as AmpC; overexpression of efflux pumps such as MexCD–OprJ, which reduces susceptibility to carbapenems; modification of PBPs, which reduces susceptibility to several β-lactams; and finally, acquisition of other β-lactamases, such as Class B carbapenemases [42]. The latter mechanism is associated with significant morbidity and mortality, as over 85% of estimated MDR *P. aeruginosa* associated infections and deaths are attributed to carbapenem resistant strains [2].

AmpC are cephalosporinases, which can hydrolyze most penicillins, early generation cephalosporins and combinations of β-lactam and β-lactamase inhibitors. They also hydrolyze aztreonam, but to a lesser degree than penicillin. What is significant about AmpC is that it is an inducible enzyme. Therefore, resistance can quickly emerge even in *P. aeruginosa* isolates that appeared to be sensitive at the beginning of treatment. Antipseudomonal cephalosporins such as ceftazidime or cefepime, while still susceptible to AmpC, are weak inducers of its expression. However, the prolonged administration of antipseudomonal β-lactams can lead to the selection of *P. aeruginosa* isolates that overproduce AmpC and subsequently to treatment failure [44,45].

ESBLs are a broad category that include various types of serine type Class A β-lactamases, such as Temoniera (TEM), cefotaxime β-lactamase (CTX-M) and sulfhydryl variable (SHV) β-lactamases. They hydrolyze penicillins, narrow and broad spectrum cephalosporins, as well as aztreonam. They cannot however hydrolyze cephamycins such as cefoxitin, or combinations of β-lactam and β-lactamase inhibitors, which can be used to differentiate ESBL positive isolates from AmpC positive isolates. Furthermore, in contrast to AmpC, ESBLs are constitutively expressed [4]. Therefore, when the presence of sensitivity to the aforementioned antibiotics on the antibiogram suggests the presence of an ESBL positive strain, the administration of antibiotics such as cefoxitin or combinations of β-lactam and β-lactamase inhibitors is unlikely to lead to the development of resistance during treatment and therapeutic failure. *P. aeruginosa* produces various Class A ESBLs, but also Class D ESBLs such as oxacillinase (OXA)-type β-lactamases, which can hydrolyze cefotaxime, ceftazidime, and aztreonam, while they are generally not inhibited by clavulanic acid [44].

Internationally, 10% to 50% of *P. aeruginosa* isolates are resistant to carbapenems. Carbapenem resistance in *P. aeruginosa* is mediated through multiple mechanisms, with porin deficiencies or efflux pump overexpression being more common, while the presence of carbapenemases is encountered less often. While *P. aeruginosa* produces serine carbapenemases, such as OXA-type carbapenemases, the majority of its carbapenemases are metallo-β-lactamases (Ambler Class B). These include imipenemase (IMP), Verona integron-encoded metallo-β-lactamase (VIM), São Paulo metallo-β-lactamase (SPM), German imipenemase (GIM) and New Delhi metallo-β-lactamase (NDM); *P. aeruginosa* is considered the main reservoir of these metalocarbapenemases [46]. Metallo-β-lactamases hydrolyze all categories of β-lactams, with the exception of aztreonam; while this may help distinguish them from other carbapenemases in the antibiogram, it is not necessarily associated with therapeutic efficacy of aztreonam in infections by these pathogens [47].

Resistance of *P. aeruginosa* to aminoglycosides is associated with a significant extent with its inborn impermeability, which renders the antibiotics incapable of penetrating its wall and reaching sufficient intracellular concentrations [48]. In addition, other intrinsic mechanisms of resistance include efflux pumps as well as aminoglycoside modifying enzymes, such aminoglycoside phosphotransferases, acetyltransferases and nucleotidyltransferases. Each of these enzymes confers resistance to different combinations of aminoglycosides, with aminoglycoside nucleotidyltransferases allowing *P. aeruginosa* to deactivate aminoglycosides commonly used against it, such as tobramycin, amikacin and gentamicin [42]. The novel aminoglycoside plazomicin has been designed precisely to overcome these mechanisms, as it is resistant to several of these enzymes [49]. Acquired mechanisms of resistance to aminoglycosides include overexpression of the MexXY efflux pump, and changes to its ribosomal target, either by mutation of the 30S ribosomal subunit or through methylation of the binding site for aminoglycosides [48,50]. Of note, ribosomal mutations can confer high-level resistance to aminoglycosides [42].

Another class of antibiotics frequently used in *P. aeruginosa* infections is fluoroquinolones. Resistance to fluoroquinolones depends on the action of efflux pumps, which are either intrinsic such as the resistance-nodulation-division (RND) family of efflux pumps, or acquired through mutations to the genes regulating the production of efflux pumps, leading to overexpression of efflux pumps such as the MexAΒ-OprM [42]. Another major mechanism is the mutation of target enzymes encoding for topoisomerase IV and DNA gyrase. Such mutations can confer a high-level of resistance to fluroquinolones, especially in the case that the genes for both enzymes are mutated or there are more than one point mutations in a single gene [51].

*P. aeruginosa* is also resistant to several other antibiotics, such as tetracyclines, macrolides, TMP/SMX and rifampicin. Production of efflux pumps confers resistance to tetracyclines and to a lesser degree to tigecycline, as the latter is an inferior substrate for the efflux pumps of *P. aeruginosa* [52]. Macrolide as well as TMP/SMX resistance are also mediated through the production of efflux pumps [53,54]. Rifampicin resistance arises through mutations in the RNA polymerase gene, although this may come at a fitness cost for *P*. *aeruginosa* [55]. Colistin is an antibiotic used as a last line of defense for MDR Gram-negative pathogens. It acts by solubilizing the cell membrane through interaction with lipid A, a key component of Gram-negative lipopolysaccharide (LPS). Colistin binds to the negatively charged phosphate groups on lipid A (which are normally bound to magnesium and calcium cations), inserts itself into the membrane and disrupts its integrity, leading to cell lysis [56,57]. Resistance to colistin in *P. aeruginosa* is based on changing the negative charge of the cell membrane, which is required for the interaction with the positively charged colistin molecule. This is mediated through the synthesis of N4-aminoarabinose, a molecule that binds to lipid A and neutralizes its negative charge [58].

## 5. Mechanisms of Antimicrobial Resistance in *E*. *coli*

Antibiotic resistant *E. coli* strains were the most common antibiotic resistant pathogen isolated in the EU during 2015, responsible for over 306,000 infections and 9828 deaths, out passing both *S. aureus* and *P. aeruginosa* combined. Of these, over 297,000 infections and 9000 deaths were caused by third-generation cephalosporin-resistant *E. coli* strains. In addition, another 7156 infections and 621 deaths were caused by colistin-resistant *E. coli* strains and an additional 2619 infections and 141 deaths were caused by carbapenem-resistant strains [2].

*E. coli* strains produce a vast array of β-lactamases, including ESBLs, AmpC and classes A, B and D carbapenemases. The first ESBLs identified in *E. coli* during the 1980s were the variants of SHV-ESBLs and TEM-ESBLs. However, the distribution of ESBLs in *E. coli* has shifted since then, with CTX-M-ESBLs becoming the predominant variant identified. Currently, the most commonly isolated ESBL gene in human isolates of *E. coli* is *blaCTX-M-15* [59]. ESBL-positive *E. coli* infections are widespread, as ESBL-positive strains made up 88.6% of the 297,000 annual infections caused by third-generation cephalosporin-resistant *E. coli* strains in the EU [2].

The other group of β-lactamases of interest in *E. coli* are the AmpC β-lactamases. *E. coli* AmpC β-lactamase was the first identified penicillinase, first reported in 1940. In contrast with most other *Enterobacteriaceae*, *E. coli* constitutively expresses low levels of AmpC β-lactamases, as the transcriptional regulator of the *ampC* gene, AmpR, is not present in *E. coli*. The regulation of AmpC occurs instead through promoter and attenuator mechanisms. Hyperproduction of AmpC occurs after mutations in the promoter or attenuator regions of the *ampC* gene, by the acquisition of a stronger promoter through insertion elements from other bacterial species and by the presence additional copies of the *ampC* gene. In addition, plasmid-mediated AmpC enzymes have also been found, but are less common than their chromosomally encoded counterparts [45,60].

The increasing resistance to third generation cephalosporins, secondary to the growing prevalence of ESBL and AmpC producing strains, has led to the increased use of carbapenems in *E. coli* infections. Inevitably, this had resulted in the emergence of carbapenem resistant strains. Although some cases of carbapenem resistance may occur through production of efflux pumps or through the combination of porin mutations with AmpC or ESBL overexpression, in the majority of cases resistance occurs through the production of carbapenemases [61]. The most prevalent carbapenemase variants in *Enterobacteriaceae* are *Klebsiella pneumoniae* carbapenemases (KPCs) of Ambler Class A, NDM, VIM and IMP of Ambler Class B, and OXA-48 of Ambler Class D. In *E. coli*, the most prevalent carbapenemases are NDM and OXA-48 [62]. It should be noted that OXA-48 has no intrinsic activity against expanded-spectrum cephalosporins such as ceftazidime and cefepime. However, this has little clinical significance, as the vast majority of isolates are resistant to these antibiotics through the production of ESBLs [63]. The number of carbapenem-resistant *E. coli* infections in the EU is on the rise, from 543 cases in 2007 to 2616 cases in 2015, while the attributable mortality has risen almost 5 times, from 29.2 deaths in 2007 to 141 deaths in 2015 [2].

The mechanisms of resistance for rifampicin, macrolides, fluoroquinolones and tetracyclines in *E. coli* are similar to those found in other *Enterobacteriaceae*. Rifampicin resistance arises through mutations in the *rpoB* gene [64]. Mechanisms of macrolide resistance include target site modification by methylases, inactivation of macrolides by esterases or phosphotransferases, as well as the production of efflux pumps. Of these, the most common mechanism in *E. coli* appears to be the production of phosphotransferases [65]. Resistance to fluoroquinolones is predominantly mediated through single-nucleotide polymorphisms in the quinolone resistance-determining region of the *gyrA* gene, while resistance to tetracyclines is mediated through the expression of efflux pumps, encoded by the *tet* genes. Tigecycline resistance has also been documented and occurs as a combination of efflux pump upregulation and porin downregulation [66,67].

Resistance to TMP-SMX, an antibiotic used extensively for community acquired UTI in the past, is now wide-spread. Mechanisms involve the overproduction of DHFR through the mutation of its promoter, or mutations in the gene of DHPS itself, which cause resistance to trimethoprim and sulfamethoxazole, respectively [54]. Aminoglycoside resistance is mediated through the production of aminoglycoside-modifying enzymes. The main enzymes encountered in *E. coli* are aminoglycoside acetyltransferases, phosphotransferases and nucleotidyltransferases. A recent study in Switzerland reported that among 3358 clinical isolates, 270 (8%) were resistant to gentamycin, 311 (9.3%) were resistant to tobramycin, while in total 470 (14%) were resistant to at least one aminoglycoside. The genotyping of 439 of the aminoglycoside-resistant strains revealed that 30.3% carried genes encoding for phosphotransferases, while another 22.8% and 11.8% carried genes for different types of acetyltransferases [68].

Resistance of *E. coli* to colistin is mediated through modifications in LPS. *E. coli* was the first pathogen in which plasmid-mediated colistin resistance was noted, through acquisition of the *mcr-1* gene [69]. Expression of MCR-1 protein leads to the addition of a phosphoethanolamine group to lipid A. This causes a change in the charge of LPS, which in turn reduces the affinity of colistin for LPS [56]. *mcr-1* has been identified so far in 32 countries including China, India, the USA and the majority of western European countries, while an additional gene for colistin resistance in *E. coli*, *mcr-2*, has been identified in Belgium [70].

## 6. Mechanisms of Antimicrobial Resistance in *K. pneumoniae*

*K. pneumoniae* is one of the most important pathogens in health care settings. Its ability to form biofilms and adhere to both surfaces and human hosts allow it to persist and spread rapidly in the environment of the hospital and colonize patients. Furthermore, its aptitude for collecting resistance plasmids has led to MDR *K. pneumoniae* becoming a worldwide problem, with some countries reporting that over half of *K. pneumoniae* clinical isolates are resistant to all available antibiotics [71,72,73]. In the EU, it is estimated that antibiotic resistant *K. pneumoniae* strains caused nearly 92,000 infections and 7500 deaths during 2015. Third-generation cephalosporin-resistant *K. pneumoniae* was responsible for 68,588 infections and 3687 deaths, whereas carbapenem and colistin resistant strains caused respectively 15,947 and 7450 infections and 2118 and 1635 deaths [2].

*K. pneumoniae* is intrinsically resistant to penicillins, such as ampicillin, through the production of several first generation β-lactamases, such as SHV-1 and TEM-1, and can acquire resistance to first- and second-generation cephalosporins through the production of broad spectrum β-lactamases (BSBL). Resistance to third- and fourth-generation cephalosporins occurs through the production of ESBLs or AmpC [4,74,75].

The most significant type of β-lactam resistance in *K. pneumoniae* is carbapenem resistance, which occurs through various mechanisms. Several types of carbapenemases can be found in *K. pneumoniae*, such as KPCs and NDM, both of which were first isolated in *K. pneumoniae*, as well as OXA-type carbapenemases. While the production of carbapenemases remains the most important mechanism of resistance, non-carbapenemase mechanisms also contribute significantly to carbapenem resistance, such as loss of outer membrane proteins and production of efflux pumps, which can also act synergistically with the overexpression of β-lactamases such as AmpC or ESBLs. The distinction between carbapenemase and non-carbapenemase mediated carbapenem resistance can be made with the modified Hodge test, in which the suspect strain is grown in the presence of a carbapenem along with a carbapenem-susceptible indicator strain; if the suspect strain produces a carbapenemase, growth of the indicator strain can be observed. It should be noted that carbapenem resistant *K. pneumoniae* infections are associated with significant mortality, which can reach 48% in hospitalized patients. Furthermore, emergence of carbapenem resistance in hypervirulent *K. pneumoniae* (strains capable of causing devastating metastatic infections including hepatic abscess and endophthalmitis) is a growing concern [76,77].

Resistance to aminoglycosides in *K. pneumoniae* occurs through the production of efflux pumps, aminoglycoside modifying enzymes that work via adenylation, acetylation or phosphorylation of target drugs, or by the production of 16SrRNA methylase, an enzyme that blocks the binding of aminoglycoside antibiotics to the 30S ribosomal subunit. In contrast to the narrow-spectrum of activity of the modifying enzymes, 16SrRNA methylase confers extremely high levels of resistance to nearly all aminoglycosides, including tobramycin, gentamicin and amikacin, which are commonly used in clinical practice [75,78].

*K. pneumoniae* has been found to express all the fluoroquinolone resistance mechanisms discovered so far in Gram-negative bacteria. These include mutations in the *gyrA* gene of the fluoroquinolone target enzyme, the overproduction of the acrAB efflux pump, the expression of the plasmid-carried *qnr* genes, which produce proteins that physically protect topoisomerase IV and DNA gyrase from fluoroquinolones, or the expression of a bifunctional aminoglycoside acetyltransferase, which in addition to aminoglycosides, can catalyze the acetylation of the piperazinyl group of norfloxacin and ciprofloxacin [79,80].

Resistance to tetracyclines occurs mainly through the expression of efflux pumps, such as tetB [81]. Resistance to tigecycline in *K. pneumoniae* mainly occurs through the overexpression of the acrAB efflux pump, which occurs either after deactivating mutations in its transcriptional repressor, AcrR, or through disinhibition of its transcriptional activator, RamA [82]. However, it should be noted that compared to tigecycline, minocycline (when sensitive) has a better in vitro antibacterial activity against KPC producing *Enterobacteriaceae*, including *Klebsiella* spp. [83,84].

Colistin resistance has also been reported in *K. pneumoniae*. Mechanisms involve the upregulation of the acrAB efflux pump, modifications to lipid A of LPS via the phosphoethanolamine transferase pathway of MCR-1 or mutations in core genes in the maturation process of lipid A, such as *lpxM* [56,79]. Rifampicin is an antibiotic with synergistic activity with colistin against KPC-producing K. pneumoniae. Resistance to rifampicin has also been reported, which occurs through the production of an ADP-ribosyltransferase [85,86].

Finally, one of the most significant attributes of *K. pneumoniae* pathogenicity is its ability to form biofilms, especially in indwelling catheters and other medical devices. Biofilms protect *K. pneumoniae* not only from host defense mechanisms, but also from antibiotics, even after prolonged exposure [87].

## 7. Mechanisms of Antimicrobial Resistance in *A. baumannii*

*A. baumannii* is a rapidly evolving nosocomial pathogen, characterized by its ability to quickly adapt to selective pressures, leading to rapid development of resistance. *A. baumannii* isolates are increasingly becoming MDR, while 30% of isolates globally are resistant to carbapenems [88,89]. Another important attribute of *A. baumannii* is its ability to survive on hospital inanimate surfaces, such as intensive care unit bed rails and equipment buttons, which serve as an important reservoir for hospital outbreaks of *A. baumannii* [90]. In the EU during 2015, infections by antibiotic resistant strains of *A. baumannii* exceeded 30,000 cases, leading to over 2500 deaths. The overwhelming majority of infections were by carbapenem-resistant *Acinetobacter* spp. (27,343 cases), 2181 infections were caused by MDR strains (resistant to aminoglycosides and fluoroquinolones), while another 1084 infections were due to colistin resistant strains [2].

*A. baumannii* produces various β-lactamases. Firstly, all *A. baumannii* strains produce a chromosomally encoded AmpC cephalosporinase, which is common to all strains. Although it is non-inducible, its production can be increased through acquisition of an insertion sequence, which contains a promoter and can lead to overexpression AmpC, conferring resistance to extended spectrum cephalospotins [89,91]. *A. baumannii* also produces various other β-lactamases, including TEM-1, CTX-M and SHV type β-lactamases [91]. Furthermore, several strains harbor ESBLs, such as TEM variants, with a reported prevalence of 59% of *A. baumannii* isolates [92].

Sulbactam, an inhibitor of several Class A β-lactamases, is an important adjuvant in *A. baumannii* infections. In addition to its activity against β-lactamases, sulbactam also inhibits PBP1 and PBP3 of *A. baumannii* (but not PBP2). While sulbactam is available only in combination with ampicillin (ampicillin-sulbactam combination in a 2:1 ratio), in vitro studies have demonstrated that the antibacterial activity of ampicillin-sulbactam against *A. baumannii* is due to the sulbactam component. In *A. baumannii* infections, sulbactam is administered in combination with carbapenems or colistin; however, high doses of ampicillin-sulbactam are required in order to achieve adequate doses of sulbactam, reaching 27 g daily [93]. High dose ampicillin-sulbactam monotherapy has been found to be comparable to colistin monotherapy in a small cohort of patients with *A. baumannii* ventilator associated pneumonia [94], while a meta-analysis concluded that regimens containing high-dose sulbactam in combination with other antibiotics provided the best chances of successful treatment in MDR *A. baumannii* infections [95]. Regardless, resistance to sulbactam can still occur in *A. baumannii*, as sulbactam is significantly less active against strains producing TEM-1 or metallo-β-lactamases, such as VIM or NDM [93].

The most significant β-lactamases produced by *A. baumannii* are the OXA-51-like Class D carbapenemases, which are characteristic of *A. baumannii*. OXA-51-like enzymes, which are penicillinases with weak carbapenemase activity, are ubiquitous in *A. baumannii* and therefore serve as a genetic identification marker for this species. *A. baumannii* also produces other OXA-type carbapenemases such as OXA-23, OXA-40 and OXA-58, found either in the bacterial chromosome or in plasmids. In addition, *A. baumannii* can also acquire potent Class B carbapenemases, such as VIM and IMP, which confer high levels of resistance against carbapenems. Furthermore, alterations in penicillin-binding proteins and porins may also contribute to carbapenem resistance [89,96].

Tetracycline resistance in *Acinetobacter* spp. occurs either through the production of ribosomal protection proteins or through the production of efflux pumps, such as TetA and TetB. Resistance to tigecycline has also been noted and is associated with the production of the AdeABC efflux pump or mutations in genes such as the *plsC* gene, which encodes for an integral membrane protein required for membrane permeability, resulting in decreased tigecycline entry to the cell, or the *trm* gene, which encodes for a methyltransferase, the reduced production of which results to reduced susceptibility to tigecycline through an unknown mechanism. However, it should be noted that AdeABC efflux pumps do not affect the susceptibility of *A. baumannii* to minocycline, and therefore even tigecycline-resistant strains can remain susceptible to minocycline [97,98,99,100]. Therefore, in the case of tigecycline resistant *A. baumannii* infections, minocycline susceptibility should always be sought.

Rifampicin is also used in *A. baumannii* infections, usually in combination with other agents, such as colistin. Rifampicin has documented synergy with colistin against *A. baumannii*, which is based on the increased penetration of rifampicin into the bacterial cell, following the effect of colistin on the Gram-negative outer membrane. Rifampicin is also synergistic with sulbactam. Resistance to rifampicin is mediated through mutations to the *rpoB* gene, or through efflux pumps, or by the enzymatic modification of rifampicin by an ADP ribosyltransferase [101,102,103].

Resistance to aminoglycosides in *A. baumannii* occurs through the production of all types of aminoglycoside modifying enzymes, including aminoglycoside acetyltransferases, nucleotidyltransferases and phosphotransferases [91]. Resistance to fluoroquinolones is mediated through mutations in the genes of DNA gyrase and topoisomerase IV, reducing their affinity for fluoroquinolones, and through the production of qnr-type protection proteins, which inhibit the binding of fluoroquinolones to topoisomerase IV and DNA gyrase. In addition, *A. baumannii* can produce efflux pumps and decrease the expression of porins, reducing intracellular concentrations of fluoroquinolones. Efflux pump production, such as the AdeABC efflux pump, confers resistance to several antibiotics at once, including cephalosporins, carbapenems, aminoglycosides, and fluoroquinolones [104].

Several mechanisms of colistin resistance have been described in *A. baumannii*, including the production of efflux pumps, the development of mutations in the genes of the initial enzymes of LPS biosynthesis resulting in the complete loss of LPS production, and alterations in the structure of lipid A. Very high levels of colistin resistance have been noted in *A. baumannii*, likely mediated through the production of efflux pumps. Colistin resistant *A. baumannii* strains also carry several other resistance genes, making their treatment extremely difficult [97,105].

## 8. Overcoming Resistance through Novel Antibiotics

**Figure 2:**
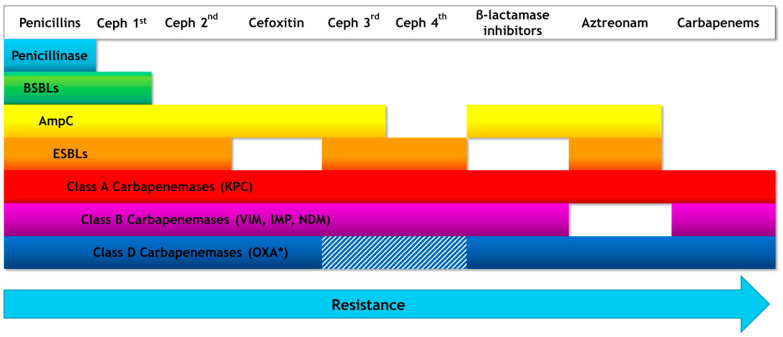
The degradation pattern for each type of β-lactamase. Cefoxitin is displayed differently from other 2nd generation cephalosporins, as hydrolysis of cefoxitin can be used to differentiate AmpC (hydrolyzes cefoxitin) from ESBLs (do not hydrolyze cefoxitin). * The different shading in the Class D carbapenemases row represents that resistance of OXA-type carbapenemases to 3rd and 4th generation cephalosporins is variable, depending on the carbapenemase variant; OXA-48 and OXA-58 have no intrinsic activity against expanded-spectrum cephalosporins, while OXA-163 and OXA-146 hydrolyze expanded-spectrum cephalosporins [63,108]. Abbreviations: Ceph: cephalosporin; BSBL: broad spectrum β-lactamase; ESBL: extended spectrum β-lactamase; KPC, *Klebsiella pneumoniae* Carbapenemase; IMP, Imipenemase type carbapenemase; NDM-1, New Delhi metallo-β-lactamase; VIM, Verona integron-encoded metallo-β-lactamase; OXA, oxacillinase.

For years, the emergence of resistant strains of bacteria was combated with the development of novel antibiotics, designed to overcome resistance. Gradually, this has led to the selection of MDR bacteria, which harbor multiple resistance genes and are impervious to several classes of antibiotics. Furthermore, resistance mechanisms targeted at newer antibiotics, often confer resistance to previously developed antibiotics in the same class, such as is the case with β-lactamases (Figure 2). While in the early 2000s the development of novel antibiotics had slowed down significantly, initiatives and incentives put in place by several organizations managed to push pharmaceutical companies towards antibiotic development [106,107]. Since then, several new antibiotics have been or are currently being developed, the majority of which belong to existing classes of antibiotics that have been modified to overcome the mechanisms that conferred resistance to their predecessors. Novel antibiotics are summarized in Table 4, along with the corresponding mechanisms they were designed to overcome.

Regarding β-lactams, recently developed antibiotics include either novel cephalosporins or novel β-lactam inhibitors which have been combined with existing β-lactams. Recently developed cephalosporins include ceftobiprole, ceftaroline, cefiderocol and ceftolozane, the latter of which is available in combination with tazobactam.

Ceftobiprole is a fifth-generation cephalosporin and is the first β-lactam to demonstrate in vitro activity against MRSA and VRSA. Ceftobiprole exhibits rapid and stable binding to PBP 2a, the altered PBP of MRSA. It is also active against PBP 2x, the PBP of penicillin resistant *Streptococcus pneumoniae*. Ceftobiprole also has an extensive Gram-negative spectrum, similar to that of third generation cephalosporins, such as ceftriaxone. However, ceftobiprole is susceptible to degradation by ESBLs, AmpC, and Class A, Class B and Class D carbapenemases. Ceftobiprole is approved for community and hospital acquired pneumonia and skin and soft-tissue infections, including diabetic foot infections [109,110].

Ceftaroline is a fifth-generation cephalosporin, distinguished by its affinity for PBP 2a, the modified PBP of MRSA that confers resistance to all β-lactams. Its spectrum of activity includes MRSA (including strains resistant to daptomycin and linezolid), VRSA, MDR *S. pneumoniae*, *Haemophilus* spp. and *Moraxella catarrhalis*. It has limited activity against enterococci, while it is inactive against Gram-negative bacteria that express ESBLs or AmpC. Ceftaroline is approved for the treatment of community-acquired pneumonia and complicated skin and soft-tissue infections [111,112].

Cefiderocol is a novel siderophore cephalosporin, which has a similar structure to ceftazidime and cefepime, with the addition of a catechol moiety. The catechol moiety allows cefiderocol to bind to free iron, mimicking the naturally occurring siderophores of Gram-negative bacteria. After binding to iron, cefiderocol is actively transported into the periplasmic space, bypassing bacterial porins, where it disassociates from iron and binds to PBPs. This allows cefiderocol to accumulate in the periplasmic space at higher concentrations than other cephalosporins. Furthermore, its unique structure makes it resistant to hydrolysis by all classes of β-lactamases, including metallo-β-lactamases; cefiderocol is stable against ESBLs, KPC (Class A), NDM, VIM, IMP (Class B), AmpC (Class C) and OXA-48 like (Class D) β-lactamases. It has been approved for complicated urinary tract infections, hospital acquired pneumonia and ventilator-associated pneumonia [113,114,115].

Ceftolozane is a novel cephalosporin with a structure similar to ceftazidime, formulated in combination with tazobactam, an established β-lactamase inhibitor. It should be noted that, while ceftolozane is hydrolyzed by ESBLs, the addition of tazobactam renders it non-susceptible to these β-lactamases. The spectrum of activity of ceftolozane-tazobactam includes several Gram-negative bacteria, including *P. aeruginosa*, *E. coli*, *K. pneumoniae*, *Burkholderia cepacia*, and *M. catarrhalis*. Ceftolozane is especially active against *P. aeruginosa*, as it is able to overcome many of the mechanisms of resistance of this bacterium, such as the production of modified PBPs or the reduction of intracellular antibiotic concentrations through alterations of porins and production of efflux pumps; it is even active against *P. aeruginosa* strains that are resistant to ceftazidime or piperacillin-tazobactam. Ceftolozane-tazobactam is approved for patients with complicated intra-abdominal and urinary tract infections, while high dose ceftolozane-tazobactam has been found to be non-inferior to meropenem in the treatment of hospital acquired pneumonia [116,117,118,119].

Recently developed β-lactam inhibitors include vaborbactam, relebactam and avibactam. Vaborbactam is a serine β-lactamase inhibitor, active against several Class A carbapenemases such as KPC (as well as ESBLs) and Class C cephalosporinases. Clinically, it is available as a combination with meropenem. Vaborbactam restores the activity of meropenem against KPC-producing organisms; however, meropenem-vaborbactam has no activity against strains producing Class B carbapenemases. This combination is approved for patients with complicated intrabdominal or urinary tract infections, hospital acquired pneumonia and ventilator associated pneumonia. Relebactam is another β-lactamase inhibitor with a similar spectrum of activity as vaborbactam. It is also active against Class A carbapenemases (KPC) and Class C cephalosporinases, while it has no activity against Class B or Class D carbapenemases. It is available as a combination with imipenem. Relebactam restores the activity of imipenem-cilastatin against carbapenem resistant *P. aeruginosa* secondary to AmpC overexpression and porin loss or against KPC-producing *Enterobacteriaceae* [120,121].

Avibactam is another novel β-lactamase inhibitor, which is active against all Class A and Class C β-lactamases, including ESBLs and KPC, as well as some class D β-lactamases. As with other novel β-lactamase inhibitors, it has no activity against metallo-β-lactamases, which are only inhibited by aztreonam, a monobactam sensitive to Class A and Class C β-lactamases, as well as some Class D β-lactamases. Therefore, the combination of aztreonam-avibactam represents an intelligent and excellent addition to the clinician’s arsenal, as it is resistant to the action of nearly all classes of β-lactamases, while it is the only available β-lactam formulation active against metallo-β-lactamase producing strains, apart from cefiderocol. Indeed, aztreonam-avibactam has been found to be active in >99% of 23,516 *Enterobacteriaceae* isolates in vitro, including strains that produced multiple serine and metallo-β-lactamases, such as VIM and NDM [122,123,124]. Avibactam has also been combined with ceftazidime, restoring the activity of ceftazidime against several carbapenemase producing *Enterobacteriaceae*. The combination of ceftazidime-avibactam has been approved for cases of complicated intrabdominal or urinary tract infections, hospital acquired pneumonia and ventilator associated pneumonia [120]. While the combination of aztreonam-avibactam is yet to receive approval, many experts recommend the co-administration of aztreonam and ceftazidime-avibactam in patients with metallo-β-lactamase producing *Enterobacteriaceae* [125].

Novel non β-lactam antibiotics are also becoming available. Delafloxacin is a novel fluoroquinolone with several distinctive characteristics. Firstly, it displays a balanced inhibition of topoisomerase IV and DNA gyrase compared to other fluoroquinolones, which may decrease the potential for development of resistance. Second, unlike other fluoroquinolones, delafloxacin has enhanced penetration and activity in the acidic environment of the infection site. Its spectrum of activity includes Gram-positive bacteria, including fluoroquinolone-resistant *S. aureus*, and Gram-negative bacteria, although its activity against *P. aeruginosa* is less than that of ciprofloxacin. Delafloxacin is approved by the Food and Drug Administration (FDA) for use in skin and soft-tissue infections and in community-acquired pneumonia [126,127].

Novel tetracyclines include omadacycline (aminomethylcycline subclass) and eravacycline (fluorocycline subclass), both of which are active against bacteria that have efflux pumps or ribosomal protection proteins, mechanisms that typically confer resistance to older tetracyclines [126,128]. Omadacycline has good oral bioavailability and a relatively long half-life (around 16 h), which permits convenient once daily dosing. It is active against MRSA, VRE, penicillin resistant and MDR *S. pneumoniae*, and is broadly active against Gram-negative bacteria, with the exception of *P. aeruginosa*. Omadacycline is approved for use in skin and soft-tissue infections and in community-acquired pneumonia [126]. Eravacycline is the first fully synthetic tetracycline. It has been found to have superior in vitro activity against several MDR pathogens compared to tigecycline, such as MDR *Acinetobacter* spp., ESBL-producing *Enterobacteriaceae*, VRE, MRSA and *S. pneumoniae*. Eravacycline is approved for complicated intra-abdominal infections. A meta-analysis of 3 randomized control trials evaluating eravacycline in complicated intra-abdominal infections found that it has similar profiles of clinical efficacy and mortality with carbapenems [128,129].

Plazomicin is a novel semisynthetic aminoglycoside that has been especially designed to withstand the action of degradation enzymes. The lack of specific hydroxyl groups protects plazomicin from inactivation by nucleotidyltransferases and phosphotransferases, while the addition of specific substitutions protects it from acetyltransferases. The end result is that plazomicin is stable against several enzymes that modify older aminoglycosides, such as gentamicin, tobramycin, and amikacin. However, resistance to plazomicin can still occur through 16SrRNA methylase, similar to other aminoglycosides. It should be noted that its activity against Gram-negative bacteria, as well as its side effect profile, does not differ from that of other aminoglycosides. Plazomicin has been approved for adults with complicated urinary tract infections [49,130].

Dalbavancin, telavancin and oritavancin are 3 novel lipoglycopeptides whthatich are utilized in the treatment of MDR Gram-positive infections. In addition to inhibition of cell wall synthesis through transglycosylation and transpeptidation, these antibiotics also display additional mechanisms of action, owing to their different modifications. The presence of lipid side chains in all 3 antibiotics enables them to anchor on the cell membrane, which increases their potency dramatically. Furthermore, oritavancin and telavancin can also disrupt the integrity, permeability and potential of the cell membrane, while oritavancin can also inhibit the synthesis of RNA. The presence of these additional mechanisms make telavancin 10 times more active than vancomycin, its parent glycopeptide. All 3 antibiotics are active against MRSA, VISA and VRE exhibiting the VanB phenotype (resistant to vancomycin but not to teicoplanin), while oritavancin is also active against VRSA and VRE exhibiting the VanA phenotype (resistant to both vancomycin and teicoplanin). This additional activity of oritavancin is due to the fact that, in addition to binding at the D-Ala-D-Ala terminal of the growing peptidoglycan chain, it can also bind to the D-Ala-D-Lac dipeptide of the modified peptidoglycan produced by the VanA operon. Dalbavancin, telavancin and oritavancin have been approved for skin and skin structure infections caused by Gram-positive organisms, while telavancin is also approved for hospital acquired and ventilator-associated pneumonia caused by *S. aureus* [131,132,133,134].

Tedizolid is a novel oxazolidinone with a structure similar to linezolid. It acts through binding to the 23S rRNA of the 50S ribosomal subunit. However, slight modifications to the molecule of tedizolid improve its potency by providing additional interactions with its binding site, while also conferring activity against linezolid-resistant strains. Compared to linezolid, tedizolid is 4 to 8 times more potent in vivo against enterococci and staphylococci, including VRE, MRSA and linezolid-resistant isolates. In addition, it has a longer half-life than linezolid, which permits once daily dosing, while retaining a high bioavailability which reaches 90%. Tedizolid is approved for skin and skin structure infections in patients over 12 years of age [135,136].

## 9. Conclusions

The exposure of bacteria to antibiotics has led to the development of resistance against every single agent utilized. Through a basic understanding the mechanisms of resistance, the clinician can better comprehend and predict resistance patterns even to antibiotics not reported on the antibiogram, and subsequently select the most appropriate antibiotic for the pathogen in question. While novel antibiotics may be able to combat established mechanisms of resistance, the unwise administration of these agents will inevitably lead to the development of resistance mechanisms against them as well.

## Figures and Tables

**Table 1:** Median number of multi-drug resistance pathogen infections and deaths in the European Union during 2015. Adapted from Cassini et al. [2].

Pathogen	Antibiotic Resistance	Median Number of Infections	Median Number of Attributable Deaths
*E. coli*	Third-Generation Cephalosporin *	297,416	9066
	Carbapenem #	2619	141
	Colistin	7156	621
	Overall	307,191	9828
*S. aureus*	Methicillin-resistant (MRSA)	148,727	7049
*P. aeruginosa*	> = 3 antibiotic groups *	9028	572
	Carbapenem #	61,892	4155
	Colistin	1262	84.5
	Overall	72,182	4811.5
*K. pneumoniae*	Third-Generation Cephalosporin *	68,588	3687
	Carbapenem#	15,947	2118
	Colistin	7450	1635
	Overall	91,985	7440
*Enterococcus* spp.	Vancomycin	16,146	1081
*Acinetobacter* spp.	Aminoglycoside and Fluoroquinolone	2182	100
	Carbapenem #	27,343	2363
	Colistin	1084	94.5
	Overall	30,609	2557.5
Overall		666,840	32,767

* Excluding isolates also resistant to colistin or carbapenem. # Excluding isolates also resistant to colistin.

**Table 2:** Summary of antibiotic resistance mechanisms in important Gram-positive bacteria.

Antibiotic Class	*S. aureus*	*Enterococcus* spp.
Penicillins	Penicillinase, production of PBP2a	Low affinity PBPs
Cephalosporins 1st gen.	PBP2a	Low affinity PBPs
Cephalosporins 2nd gen.	PBP2a	Low affinity PBPs
Cephalosporins 3rd gen.	PBP2a	Low affinity PBPs
Cephalosporins 4th gen.	PBP2a	Low affinity PBPs
b-lactamase inhibitors	PBP2a	
Carbapenems	Development of PBP2a	Low affinity PBPs
Tetracyclines	Ribosomal methylation of binding sites, efflux pumps	Ribosomal methylation of binding sites, efflux pumps
Tigecyclines	Efflux pumps	Ribosomal methylation of binding sites, efflux pumps
Macrolides and clindamycin	Ribosomal methylation of binding sites, efflux pumps	Efflux pumps, clindamycin inactivating enzymes
Fluoroquinolones	Mutations in topoisomerase IV and DNA gyrase, efflux pumps	Mutations in topoisomerase IV and DNA gyrase, production of protection proteins
Rifampicin	Mutations in RNA polymerase gene	Mutations in RNA polymerase gene
TMP/SMX	Mutations in DHPS and DHFR	Folate absorption from environment
Aminoglycosides	Aminoglycoside degradation enzymes	Aminoglycoside degradation enzymes, ribosomal mutations
Daptomycin	Electrostatic repulsion through increase to the cell-surface charge	*E faeccium*: electrostatic repulsion through increase to the cell-surface charge. *E. faecalis*: redistribution of cardiolipin away from septum plane
Vancomycin	VRSA: altered structure of peptidoglycan precursors from D-Ala-D-Ala to D-Ala-D-Lac; VISA: increased production of peptidoglycan, thicker cell wall, decoy D-Ala-D-Ala dipeptides on cell surface	Altered structure of peptidoglycan precursors from D-Ala-D-Ala to D-Ala-D-Lac
Linezolid	Mutations to the 23S rRNA, altering required modifications to the 23S rRNA, mutations to the 50S ribosomal L3 protein	Mutations to the 23S rRNA

Abbreviations: PBP, penicillin binding protein; TMP/SMX, trimethoprim-sulfamethoxazole; DHPS, dihydropteroate synthase; DHFR, dihydrofolate reductase.

**Table 3:** Summary of antibiotic resistance mechanisms in important Gram-negative bacteria.

Antibiotic Class	*P. auruginosa*	*E. coli*	*K. pneumoniae*	*A. baumanii*
Penicillins	AmpC, ESBLs, other b-lactamases	AmpC, ESBLs, other b-lactamases	AmpC, ESBLs, other b-lactamases	AmpC, ESBLs, other b-lactamases
Cephalosporins 1st gen.	AmpC, ESBLs	AmpC, ESBLs	AmpC, ESBLs	AmpC, ESBLs
Cephalosporins 2nd gen.	AmpC, ESBLs	AmpC, ESBLs	AmpC, ESBLs	AmpC, ESBLs
Cephalosporins 3rd gen.	AmpC, ESBLs	AmpC, ESBLs	AmpC, ESBLs	AmpC, ESBLs
Cephalosporins 4th gen.	ESBLs	ESBLs	ESBLs	ESBLs
b-lactamase inhibitors	AmpC	AmpC	AmpC	AmpC
Aztreonam	ESBLs	ESBLs	ESBLs	ESBLs
Carbapenems	Class B & D carbapenemases	Class A, B & D carbapenemases	Class A, B & D carbapenemases	Class B & D carbapenemases
Tetracyclines	Efflux pumps	Efflux pumps	Efflux pumps	Efflux pumps
Tigecycline	Efflux pumps	Efflux pumps, porin downregulation	acrAB efflux pump	AdeABC efflux pump, reduced membrane permeability
Macrolides and clindamycin	Efflux pumps	Efflux pumps, macrolide inactivating enzymes, target site modification		
Fluoroquinolones	Mutations in topoisomerase IV and DNA gyrase genes, efflux pumps	Mutations in DNA gyrase gene	Mutations in DNA gyrase gene, efflux pumps, enzyme protection proteins, fluoroquinolone degradation enzymes	Mutations in genes for DNA gyrase and topoisomerase IV, efflux pumps, enzyme protection proteins, fluoroquinolone degradation enzymes
Rifampicin	Mutations in RNA polymerase gene	Mutations in RNA polymerase gene	Enzymatic degradation	Mutations in RNA polymerase gene, efflux pumps, enzymatic degradation
TMP/SMX	Efflux pumps	Overproduction of DHFR, mutation of DHPS		
Aminoglycosides	Aminoglycoside degradation enzymes, efflux pumps	Aminoglycoside degradation enzymes	Aminoglycoside degradation enzymes, production of 16SrRNA	Aminoglycoside degradation enzymes
Colistin	Reduction of membrane negative charge through addition of N4-aminoarabinose to lipid A	Reduction of membrane negative charge through addition of phosphoethanolamine to lipid A	Reduction of membrane negative charge through addition of phosphoethanolamine to lipid A	efflux pumps, loss of LPS production, alterations in the structure of lipid A

Abbreviations: ESBLs, extended-spectrum-β-lactamases; TMP/SMX, trimethoprim-sulfamethoxazole; DHPS, dihydropteroate synthase; DHFR, dihydrofolate reductase.

**Table 4:** Novel antibiotics, mechanisms of overcoming resistance and spectrum of activity.

Antibiotic	Resistance Mechanisms Designed to Overcome	Active Against	Inactive Against	Indications
Cephalosporins				
Ceftobiprole	Active against altered PBPs, such as PBP2a and PBP2x	MRSA, VRSA, PRSP, Gram-negative bacteria	ESBLs, AmpC, Class A, B and D carbapenemases	CAP, SSTI
Ceftaroline	Active against altered PBPs, such as PBP2a	MRSA	ESBLs, AmpC, Class A, B and D carbapenemases	CAP, SSTI
Cefiderocol	Utilizes iron to bypass porins, accumulates in periplasmic space. Resistant to hydrolysis to all β-lactamases: ESBLs, AmpC, Class A, B and D carbapenemases	MDR Gram-negative bacteria	-	HAP, VAP, cUTI
Ceftolozane-tazobactam	Ceftolozane overcomes *P. aeuruginosa* resistance mechanisms: efflux pumps, altered PBPs and porins. Tazobactam confers resistance to ESBLs	MDR *P. aeuruginosa*		cUTI, cIAI
Novel β-lactam inhibitor combination				
Meropenem-vaborbactam	Vaborbactam inhibits ESBLs, Class C cephalosporinases and Class A carbapenemases	MDR Gram-negative bacteria	Class B carbapenemases	HAP, VAP, cUTI, cIAI
Imipenem-relebactam	Relebactam inhibits ESBLs, Class C cephalosporinases and Class A carbapenemases	MDR Gram-negative bacteria	Class B and D carbapenemases	
Aztreonam-avibactam	Avibactam inhibits all Class A and Class C, and some Class D β-lactamases. Aztreonam inhibits Class B β-lactamases.	MDR Gram-negative bacteria	-	Approval pending
Ceftazidime-avibactam	Avibactam inhibits all Class A and Class C, and some Class D β-lactamases.	MDR Gram-negative bacteria	Class B carbapenemases	HAP, VAP, cUTI, cIAI
Fluoroquinolones				
Delafloxacin	Balanced inhibition of topoisomerase IV and DNA gyrase, decreasing resistance potential. Enhanced penetration and activity in acidic environments, such as infection sites	Fluoroquinolone-resistant *S. aureus*, Gram-negative bacteria		CAP, SSTI
Tetracyclines				
Omadacycline	Active against tetracycline efflux pumps or ribosomal protection proteins	MRSA, VRE, PRSP, Gram-negative bacteria	*P. aeruginosa*	CAP, SSTI
Eravacycline	Active against bacteria that have efflux pumps or ribosomal protection proteins	MDR *Acinetobacter* spp., ESBL-producing *Enterobacteriaceae*, VRE, MRSA and *S. pneumoniae*		cIAI
Aminoglycoside				
Plazomicin	Resistant to degradation by aminoglycoside nucleotidyltransferases, phosphotransferases and acetyltransferases	Gram-negative bacteria that produce aminoglycoside degradation enzymes	16SrRNA methylase producing bacteria	cUTI
Lipoglycopeptides				
Dalbavancin	Increased membrane anchoring	MRSA, VISA and VRE exhibiting the VanB phenotype	VRSA and VRE exhibiting the VanA phenotype	SSTI
Telavancin	Increased membrane anchoring, disruption of membrane integrity, permeability and potential	MRSA, VISA and VRE exhibiting the VanB phenotype	VRSA and VRE exhibiting the VanA phenotype	SSTI, HAP & VAP by *S. aureus*
Oritavancin	Increased membrane anchoring, disruption of membrane integrity, permeability and potential, RNA synthesis inhibition, binding to both D-Ala-D-Ala & D-Ala-D-Lac dipeptides	MRSA, VISA & VRE with VanB phenotype, VRSA & VRE with VanA phenotype	-	SSTI
Oxazolidinone				
Tedizolid	More potent binding to the 23S rRNA binding site than linezolid	VRE, MRSA and linezolid-resistant isolates	-	SSTI

Abbreviations: CAP, community acquired pneumonia; SSTI, skin and soft tissue infections; HAP, hospital acquired pneumonia; VAP, ventilator associated pneumonia; cUTI, complicated urinary tract infections; cIAI, complicated intrabdominal infections; ESBLs, extended-spectrum-β-lactamases; PBP, penicillin binding protein; PRSP, penicillin resistant S. penumoniae.
